# Hypoxia-induced complement component 3 promotes aggressive tumor growth in the glioblastoma microenvironment

**DOI:** 10.1172/jci.insight.179854

**Published:** 2024-08-22

**Authors:** Rebecca Rosberg, Karolina I. Smolag, Jonas Sjölund, Elinn Johansson, Christina Bergelin, Julia Wahldén, Vasiliki Pantazopoulou, Crister Ceberg, Kristian Pietras, Anna M. Blom, Alexander Pietras

**Affiliations:** 1 Division of Translational Cancer Research, Department of Laboratory Medicine, Lund University Cancer Centre, Lund University, Lund, Sweden.; 2 Section of Medical Protein Chemistry, Department of Translational Medicine, Lund University, Malmö, Sweden.; 3 Division of Medical Radiation Physics, Department of Clinical Sciences, Lund University, Lund, Sweden.

**Keywords:** Oncology, Cancer, Complement, Hypoxia

## Abstract

Glioblastoma (GBM) is the most aggressive form of glioma with a high rate of relapse despite intensive treatment. Tumor recurrence is tightly linked to radio-resistance, which in turn is associated with hypoxia. Here, we discovered a strong link between hypoxia and local complement signaling using publicly available bulk, single-cell, and spatially resolved transcriptomic data from patients with GBM. Complement component 3 (*C3*) and the receptor *C3AR1* were both associated with aggressive disease and shorter survival in human glioma. In a genetically engineered mouse model of GBM, we found C3 specifically in hypoxic tumor areas. In vitro, we found an oxygen level–dependent increase in *C3* and *C3AR1* expression in response to hypoxia in several GBM and stromal cell types. C3a induced M2 polarization of cultured microglia and macrophages in a C3aR-dependent fashion. Targeting C3aR using the antagonist SB290157 prolonged survival of glioma-bearing mice both alone and in combination with radiotherapy while reducing the number of M2-polarized macrophages. Our findings establish a strong link between hypoxia and complement pathways in GBM and support a role of hypoxia-induced C3a/C3aR signaling as a contributor to glioma aggressiveness by regulating macrophage polarization.

## Abstract 2

Glioblastoma, highly resistant to treatment, shows strong links between hypoxia and complement signaling, impacting macrophage polarization, and suggesting novel therapeutic targets.

## Graphical abstract

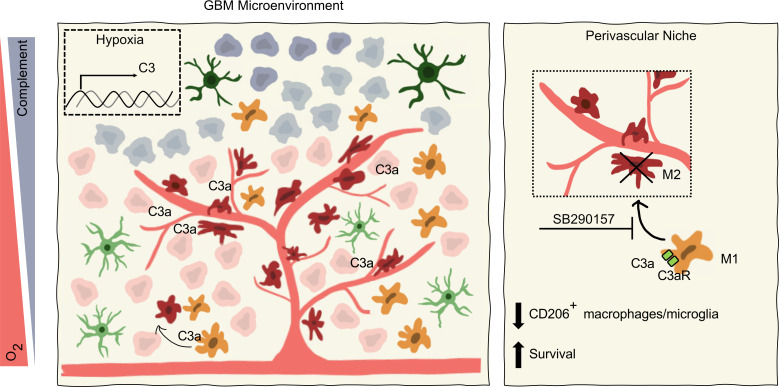

## Introduction

Glioblastoma (GBM) is the most common and aggressive primary brain tumor in adults (1). Despite intensive treatment with surgery, irradiation, and chemotherapy, all patients suffer recurrence of treatment-resistant tumors (2, 3). The overall survival for patients remains low, with a median survival of less than 2 years (4). GBM is characterized by abundant pseudopalisading necroses, which are surrounded by areas of severe hypoxia (5). Tumor hypoxia is associated with tumor angiogenesis and a metabolic shift toward anaerobic glycolysis, as well as tumor cell stemness and radioresistance (6–8). Although the malignant cell response to hypoxia is well characterized, heterotypic cell-cell signaling involving stromal cells in the hypoxic niche remains poorly understood.

Prominent nonmalignant cell types including microglia/macrophages (9), astrocytes (10), and endothelial cells (11) play tumor-supportive roles in the GBM microenvironment. Within the tumor, stress related to, for example, hypoxia, chemotherapy, and radiotherapy is not restricted to the tumor cell compartment but is likely to affect the phenotype and behavior of the entire spectrum of cell types in the microenvironment. Indeed, we have previously shown that radiotherapy results in a net tumor-supportive microenvironment in the brain (12, 13). Specifically, tumor-associated astrocytes respond to radiotherapy (12) and severe hypoxia (14) with a reactive phenotype that, in turn, promotes aggressive tumor growth. Similarly, radiotherapy results in an altered immune environment in part because of radiation-induced phenotypic shifts of microglia and macrophages (15). It is likely that hypoxia-induced gene expression changes result in altered cell-cell communication between stromal and tumor cells.

Complement component 3 (C3) is a key marker of astrocyte reactivity (16–18) and a central component of the complement system of innate immunity, found at high levels in serum. Most circulating complement proteins from serum are restricted from entering the brain due to the blood-brain barrier (BBB); thus, most complement proteins in the brain are expressed locally or enter through a leaky BBB (19, 20). When activated by proteolysis, C3 cleavage products, including C3a, can signal via various receptors, affecting cellular activation, inflammation, and metabolism (21). However, its presence and function in tumor-associated gliosis remains undetermined. C3a/C3aR signaling has been implicated in brain development, neural plasticity, and protection against hypoxic-ischemic brain injuries (22–24), suggesting the potential of a role also in the setting of brain tumors. Here, we used publicly available transcriptomic data, a genetically engineered mouse model of GBM, and primary cultures of GBM and stromal cell types to investigate the presence and function of C3 in the GBM microenvironment as well as its role in relation to hypoxia.

## Results

### C3 is associated with aggressiveness in glioma.

**Figure 1:**
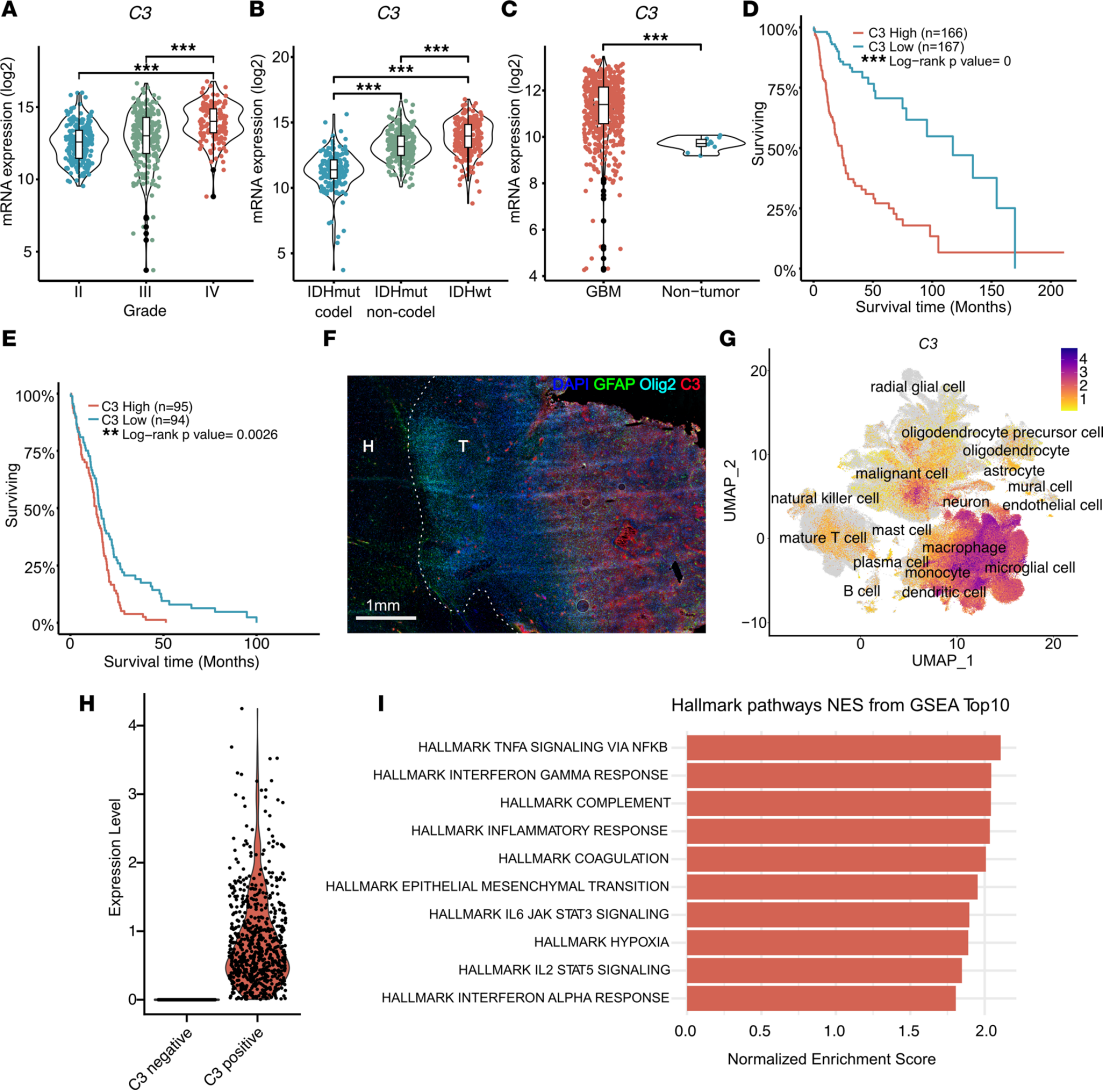
C3 is associated with aggressive GBM. (**A**) TCGA data analyzed for *C3* expression in glioma grades II, III, and IV. (**B**) TCGA data analyzed for *C3* expression in IDH-WT (IDHwt) glioma compared with IDH mutant (IDHmut) with or without 1p/19q codeletion. (**C**) TCGA data analyzed for *C3* expression in GBM compared with nontumor. (**D**) Kaplan-Meier curve showing survival of glioma patients with either high (red) or low (blue) C3 expression based on TCGA data. (**E**) Kaplan-Meier curve showing survival of IDHwt GBM with high (red) or low (blue) *C3* expression based on TCGA data. (**F**) Murine GBM stained for C3, Olig2, GFAP, and DAPI as indicated. T indicates tumor, and H indicates healthy brain tissue. (**G**) UMAP displaying *C3* expression in single-cell sequencing data from 16 independent data sets compiled in GBmap (25). (**H**) Malignant cell population divided into *C3*^–^ (86.4%) or *C3*^+^ (13.6%) cells. (**I**) Gene set enrichment signature pathways associated with *C3* expression in malignant cells. Red bars indicate significant Benjamini-Hochberg adjusted *P* values (*P*_adj_ < 0.05). *P < 0.05, **P < 0.01, or ***P< 0,001. Statistical tests were 1-way ANOVA, or unpaired *t* test (in case of comparison between 2 groups) with Tukey post hoc test.

Bulk RNA-Seq from TCGA data sets on GBM and low-grade glioma (TCGA_GBM and TCGA_GBMLGG) showed that *C3* expression increased with glioma grade (Figure 1A) and IDH-WT status (Figure 1B) and was expressed at higher levels in GBM compared with nontumor brain (Figure 1C). *C3* expression correlated with worse survival in patients with glioma (Figure 1D), as well as in isocitratedehydrogenase-WT (IDH-WT) GBM (Figure 1E). We generated murine GBM using the replication-competent avian sarcoma-leukosis virus long terminal repeat with splice acceptor/tumor virus A (RCAS/tv-a) system to express *PDGFB* and shRNA targeting *Tp53* in Nestin-expressing cells of the neonatal Nestin/tv-a (Ntv-a) mouse brain. Tumors were stained for C3, GFAP as an astrocytic marker, and Olig2 for the bulk of tumor cells. In line with TCGA data demonstrating higher levels of *C3* in GBM as compared with nontumor brain tissue (Figure 1C), we found the vast majority of C3 present within the tumor core, with virtually no detectable stain in the tumor-adjacent healthy brain tissue (Figure 1F).

We next queried the highly integrated GBmap from Ruiz-Moreno et al. (25), comprising 16 independent single-cell RNA-Seq data sets, and a total of over 330,000 cells from 110 patients with GBM (Supplemental Figure 1A; supplemental material available online with this article; https://doi.org/10.1172/jci.insight.179854DS1) (25). Analysis showed that *C3* is expressed by several cell types, including macrophages, microglia, astrocytes, endothelial, T cells, and tumor cells (Figure 1G). We classified cells within each cell type into either *C3*^–^ or *C3*-expressing cells (Figure 1H and Supplemental Figure 1, B–D) and asked whether certain gene sets were associated with *C3* expression. Gene set enrichment analysis (GSEA) revealed that *C3*-expressing cells in all tested cell types (astrocytes [Supplemental Figure 1, B and E], mural cells [Supplemental Figure 1, C and F], and macrophages/microglia [Supplemental Figure 1, D and G]) showed an enrichment for gene signatures associated with inflammatory processes such as TNF-α and IFN-γ signaling (Figure 1I). Interestingly, *C3*-expressing malignant cells were associated with gene sets for EMT and hypoxia (Figure 1I). Based on the entire data set, cells that scored positive for *C3* expression showed increased expression of several genes involved in activation of the classical complement pathway (*C1QA*, *C1QB*, *C1QC*, and *C3*), as well as Complement Factor D (*CFD*) from the alternative complement pathway (Table 1). Early complement inhibitory genes *CD46*, *CD55*, and *CSMD1* were downregulated (Table 1). Interestingly, late complement inhibitors such as *CD59* were also increased in *C3*-expressing cells, suggesting that *C3*-expressing cells may still be protected from cellular lysis mediated through the membrane attack complex (MAC), while there is a potential for early complement activation to take place (Table 1).

### Hypoxia is associated with local complement signaling in GBM tumors.

**Figure 2:**
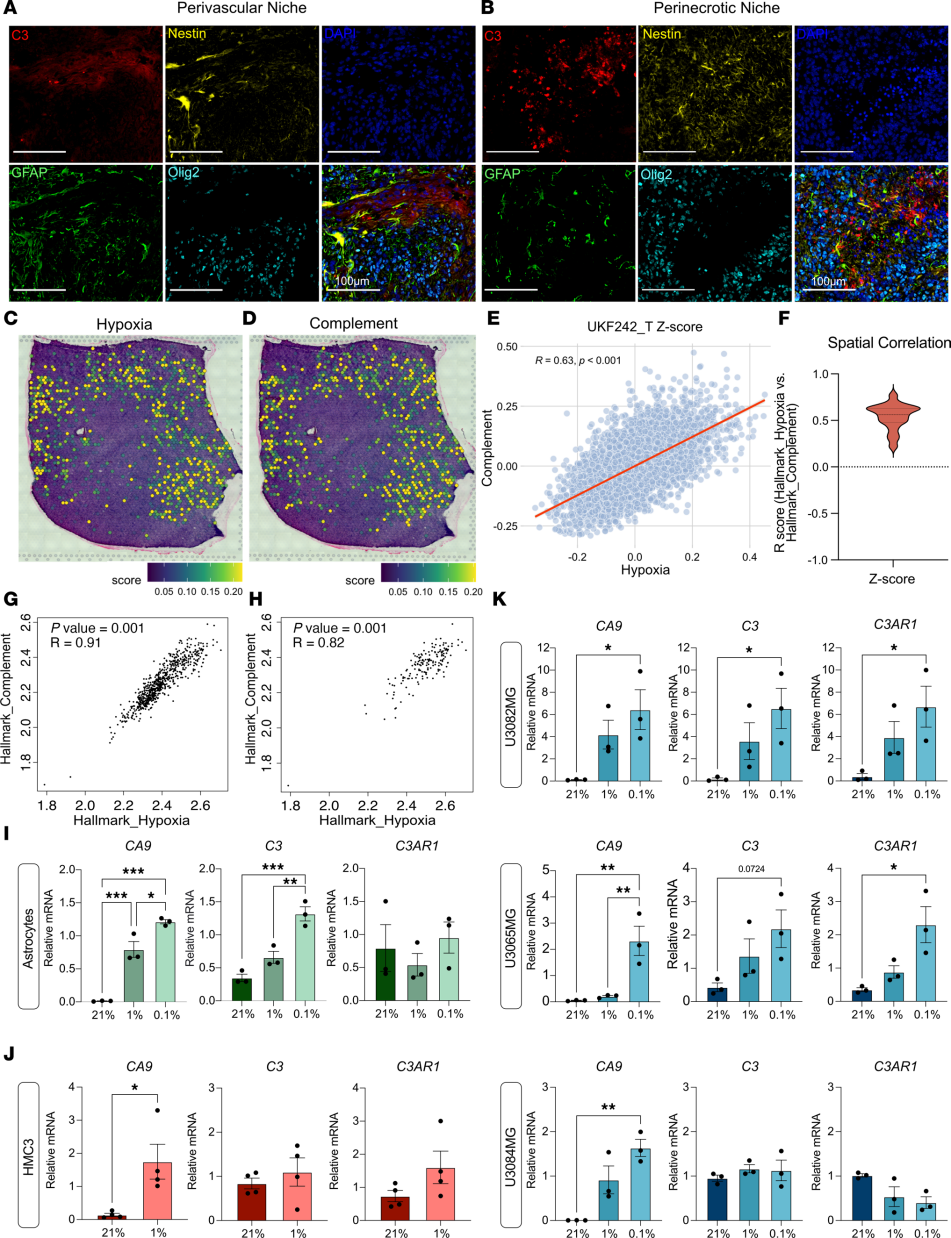
Hypoxia is associated with local complement signaling in GBM tumors. (**A** and **B**) Immunofluorescence detection of C3 (red), Nestin (yellow), Nuclei (DAPI, blue), GFAP (green), or Olig2 (cyan) in the perivascular (**A**) or hypoxic (**B**) niches of murine RCAS-PDGFB– and RCAS-shp53–induced gliomas. (**C** and **D**) HALLMARK_HYPOXIA (**C**) and HALLMARK_COMPLEMENT (**D**) signatures mapped on spatially resolved transcriptomics from human GBM samples, displayed with *Z* score. (**E**) Spatial correlation between HALLMARK_HYPOXIA and HALLMARK_COMPLEMENT in 1 representative tumor (UKF242). *P* values were corrected for multiple hypothesis testing using the Holm method. (**F**) Distribution of R values for the correlation between HALLMARK_HYPOXIA and HALLMARK_COMPLEMENT in a total of 19 human GBM tissue samples with an average correlation score of R = 0.54 (26). (**G** and **H**) Pearson correlation coefficient between HALLMARK_COMPLEMENT and HALLMARK_HYPOXIA signatures in the TCGA GBMLGG (**G**) or TCGA GBM (**H**) data set. (**I**–**K**) Expression of *CA9*, *C3*, and *C3AR1* mRNA in human primary astrocytes (*n* = 3), HMC3 microglia (*n* = 4), or primary human glioma cells U3082MG (*n* = 3), U3065MG (*n* = 3), or U3084MG (*n* = 3) in response to normoxia (21% O_2_), hypoxia (1% O_2_), or severe hypoxia (0.1% O_2_). Data are shown as mean ± SEM. *P < 0.05, **P < 0.01, or ***P< 0,001. Statistical tests were 1-way ANOVA, with Tukey post hoc test, or unpaired *t* test in case of 2 sample comparisons. Statistical tests were 1-way ANOVA (**I** and **K**), with Tukey post hoc test, or unpaired *t* test in case of 2-sample comparisons (**J**).

To understand the role of C3 and complement in the tumor microenvironment, we returned to stain the murine GBMs (Figure 1F) for C3, GFAP, Olig2, and Nestin to highlight perivascular tumor areas. C3 was present primarily in perivascular (Figure 2A) and perinecrotic (hypoxic) (Figure 2B) tumor niches, in close proximity to Nestin-expressing cells. Considering the high C3 levels found in hypoxic tumor areas and the association between *C3*-expressing GBM cells and the hypoxia gene signature (Figure 1I), to further investigate the relationship of complement expression to hypoxia, we applied the HALLMARK_COMPLEMENT and HALLMARK_HYPOXIA gene expression signatures to a publicly available data set of spatial transcriptomics from 19 patients with GBM (26). These analyses revealed a strong spatial correlation of the 2 signatures (Figure 2, C–F), with the complement signature almost exclusively present in tumor areas scoring high for the hypoxia signature. Bulk RNA-Seq from TCGA confirmed a strong correlation of the signatures in both low- (Figure 2G) and high-grade gliomas in larger patient cohorts (Figure 2H). In the human GBM single-cell data set (GBmap), we found that the hypoxia (Supplemental Figure 1B) and complement (Supplemental Figure 1C) signatures are primarily expressed by cells derived from the myeloid linage, such as macrophages, monocytes, and microglial cells, as well as endothelial cells, astrocytes, and — to some extent — tumor cells themselves in human GBM (Supplemental Figure 2, A–D). We next sought to determine whether *C3* is directly upregulated in response to hypoxia. Astrocytes, microglia, and human primary GBM cells were cultured in various oxygen tensions (normoxia [21% O_2_], hypoxia [1% O_2_], and severe hypoxia [0.1% O_2_]) for 72 hours. Hypoxia induction was confirmed by increased levels of *CA9* (Figure 2, I–K). *C3* expression was significantly induced by hypoxia in astrocytes and 2 of 3 GBM cell lines, and *C3AR1* expression was significantly induced in 2 of 3 GBM cell lines tested (Figure 2, I–K). Together, these data show that local C3 expression in GBM is highly associated with hypoxia and that growth under hypoxic conditions themselves can cause upregulation of *C3* and *C3AR1* in some cells.

### Limited effects of C3 on proliferation and survival of glioma cells.

**Figure 3:**
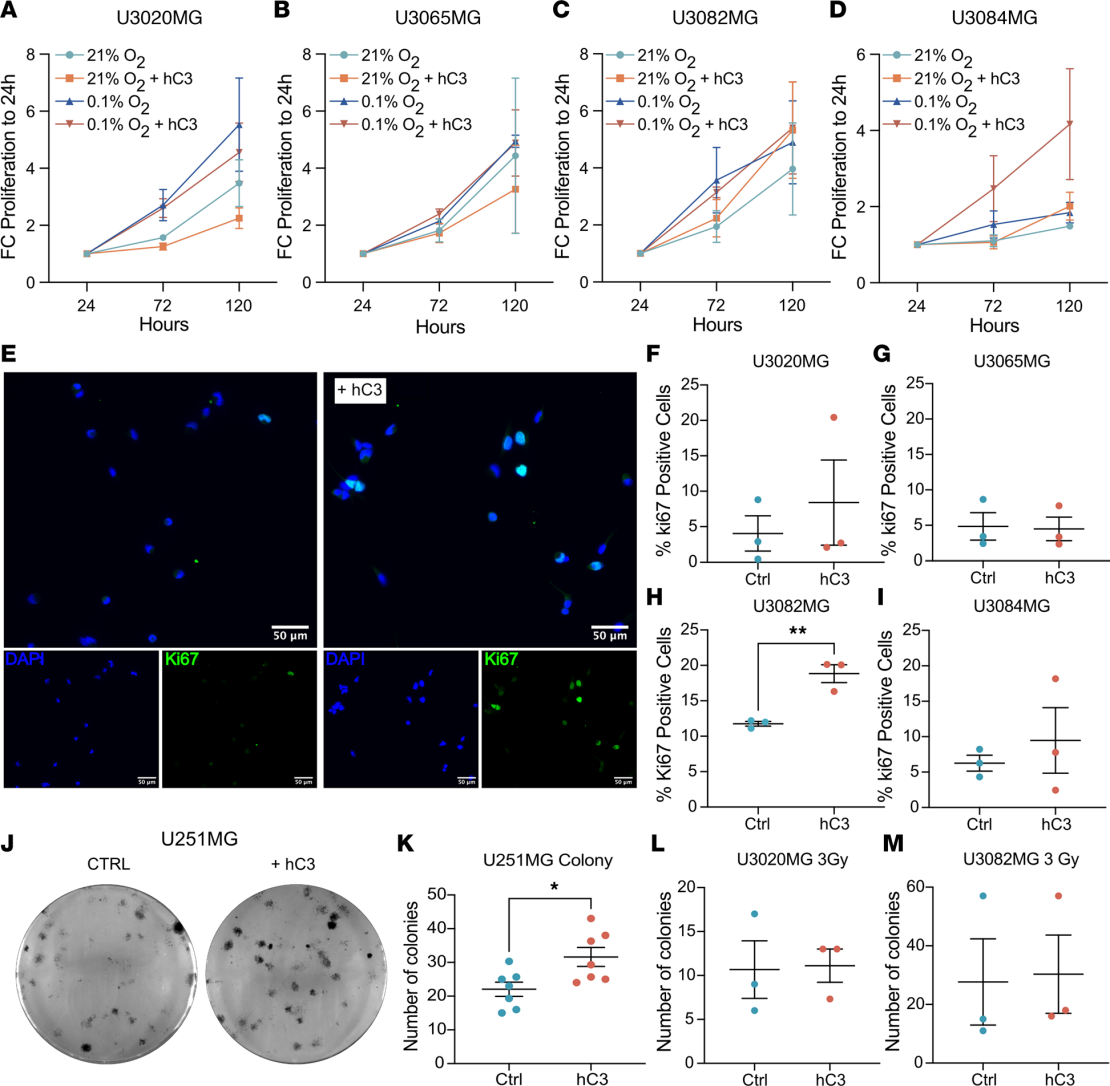
Limited effects of C3 on glioma cell growth under stressful conditions. (**A**–**D**) U3082MG (*n* = 5), U3065MG (*n* = 3), U30884MG (*n* = 3), and U3020MG (*n* = 3) glioma cell proliferation under normoxic (21% O_2_) and severe hypoxic (0.1% O_2_) conditions with or without the presence of human serum-purified C3 as measured by the WST-1 assay. (**E**) Representative images of immunofluorescence detection of Ki67^+^ cells in U3082MG glioma cells grown under severe hypoxia (0.1% O_2_) with or without the presence of C3 (180 ng/mL). Scale bars: 50 µm. (**F**–**I**) Quantification of Ki67^+^ cells (*n* = 3). (**J**) Representative image of clonal survival of U251MG glioma cells after 4 Gy irradiation with or without presence of human C3 (180 ng/mL) in the medium in triplicate wells. (**K**–**M**) Quantification of number of colonies in U251MG (*n* = 7), U3020MG (*n* = 3), and U3082MG (*n* = 3) cells. Data are shown as mean ± SEM. *P < 0.05, **P < 0.01 from unpaired *t* tests.

To determine whether presence of C3 can modulate tumor cell properties, we added serum-purified human C3 in serum-free culture medium of U3082MG, U3084MG, U3020MG, and U3065MG primary glioma cells. Proliferation rates were not consistently affected by C3 in any of the tested cell lines (Figure 3, A–D); however, the proportion of Ki67^+^ cells (Figure 3, E–I) increased in presence of C3 specifically under hypoxic conditions, when *C3AR1* was upregulated (Figure 2K), in U3082MG cells but not in the other tested cultures. In a clonal survival assay following a single dose of 3–4 Gy irradiation, U251MG glioma cells formed significantly more colonies when C3 was present in the culture medium (Figure 3, J and K); however, this effect was not apparent in U3020MG or U3082MG cells, either with or without radiation treatment (Figure 3, L and M, and Supplemental Figure 3). Together, these data suggest a limited role for C3 as a prosurvival factor for GBM cells during cellular stress such as hypoxia and irradiation (27, 28).

### C3AR1 is highly expressed in GBM and is associated with aggressive tumor growth.

**Figure 4:**
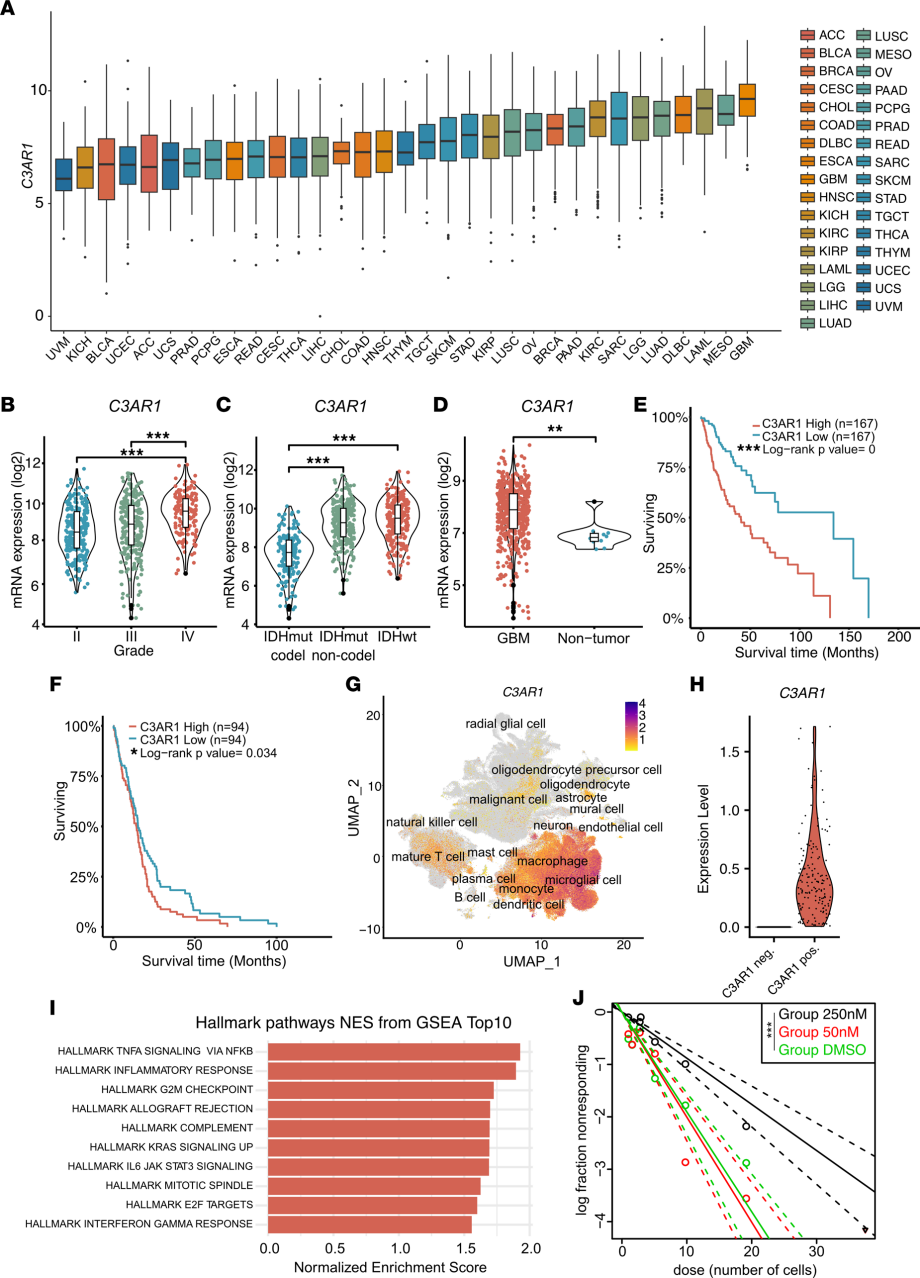
*C3AR1* is associated with aggressive GBM. (**A**) *C3AR1* expression of Pan-Cancer TCGA data of common cancer types (*n* = 33). (**B**) *C3AR1* expression in relation to glioma grade as analyzed in TCGA data. (**C**) *C3AR1* expression in IDHwt glioma compared with IDHmut with or without 1p/19q codeletion (Tukey post hoc test) as analyzed in TCGA data. (**D**) *C3AR1* expression in GBM compared with nontumor as analyzed in TCGA data. (**E**) Kaplan-Meier curve showing survival of patients with glioma with either high (red) or low (blue) *C3AR1* expression based on TCGA data. (**F**) Kaplan-Meier curve showing survival of IDHwt GBM with high (red) or low (blue) *C3AR1* expression based on TCGA data. (**G**) UMAP displaying *C3AR1* expression in single-cell RNA-Seq data from 26 independent data sets compiled in GBmap (25). (**H**) *C3AR1* expression of malignant cells divided into *C3AR1*^+^ (3.1%) or *C3AR1*^–^ (96.9%) cells. (**I**) GSEA of the *C3AR1*-expressing malignant cells. Red bars indicate significant Benjamini-Hochberg adjusted *P* values (*P*_adj_ < 0.05). (**J**) Log-fraction plot of a combination of independent extreme limiting dilution sphere formation assays (*n* = 4) of U3082MG glioma cells treated with SB290157. *P < 0.05, **P < 0.01, or ***P< 0,001. One-way ANOVA (**B** and **C**), or unpaired *t* test (**D**) (in case of comparison between 2 groups) with Tukey post hoc test.

We next sought to investigate the expression of complement receptors in GBM using TCGA data. We found that *C3AR1* expression is higher in GBM as compared with all other cancers analyzed in TCGA (Figure 4A), whereas Complement Receptor 1 (*CR1*), *CR2*, C1qR1 (*CD93*), and *C5AR1* were expressed at varying levels (Supplemental Figure 4, A–D). C1qR1 (*CD93*) (Supplemental Figure 4E), *C3AR1* (Figure 4B), and *C5AR1* (Supplemental Figure 4F), but not *CR1* (Supplemental Figure 4G) or *CR2* (Supplemental Figure 4H), were highly upregulated in GBM compared with normal brain tissue. *C3AR1* increased with glioma grade (Figure 4B) and IDH-WT status (Figure 4C) and was expressed at higher levels in GBM as compared with nontumor brain (Figure 4D). Furthermore, *C3AR1* was associated with shorter survival in glioma (Figure 4E) as well as in IDH-WT GBM (Figure 4F). Single-cell RNA-Seq data from GBmap show that *C3AR1* was expressed mainly by cells of the immune compartment, such as microglia/macrophages and mature T cells (Figure 4G). Furthermore, the tumor cells that were positive for *C3AR1* scored high for signatures associated with inflammation, as well as several pathways associated with cell cycle regulation (Figure 4, H and I). Because of the high expression of *C3AR1* in GBM, we tested the effect of the C3aR antagonist SB290157 in the extreme limiting dilution assay (ELDA) — a functional assay measuring the self-renewal capability of U3082MG glioma cells. U3082MG cells treated with SB290157 showed a reduced ability to form spheres (Figure 4J), suggesting that signaling through C3aR contributes to glioma cell self-renewal.

### C3a promotes M2-like polarization of microglia and macrophages.

**Figure 5:**
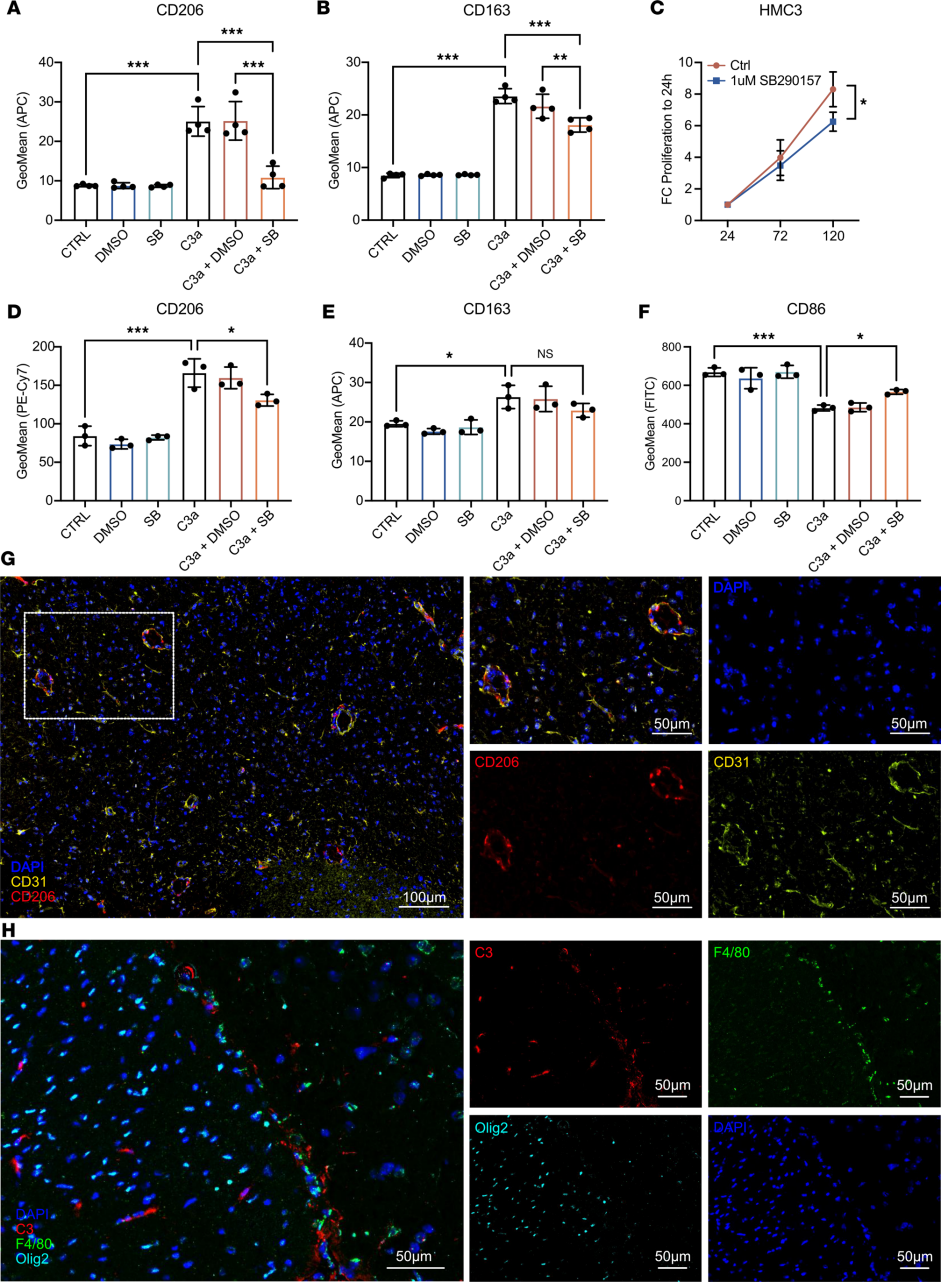
C3a promotes M2 polarization of microglia and macrophages. (**A** and **B**) Flow cytometry for indicated markers of M2 polarization in HMC3 microglia cells treated as indicated with SB290157 (SB), DMSO, and C3a (*n* = 4). (**C**) Proliferation of HMC3 cells treated or not with SB290157 as measured by the WST-1 assay (*n* = 3). (**D**–**F**) Flow cytometry for indicated markers of M2 (CD206, CD163) and M1 (CD86) polarization in primary murine macrophages treated as indicated with SB290157 (SB), DMSO, and C3a (*n* = 3). (**G**) Representative image of immunofluorescence staining of CD31 and CD206 in murine gliomas induced through RCAS-PDGFB and RCAS-shp53 in Nestin/tv-a mice. Scale bars: 100 μm, 50 μm (insets). (**H**) Representative image of immunofluorescence staining of C3 and F4/80 in murine gliomas induced through RCAS-PDGFB and RCAS-shp53 in Nestin/tv-a mice. Scale bars: 50 μm.

Given the high *C3* and *C3AR1* expression in myeloid cells, we next tested the effect of C3a on polarization and growth of cultured microglia and macrophages. Human HMC3 microglia cells displayed increased expression of CD206 and CD163 — both markers of M2-polarized macrophages — by flow cytometry when cultured with C3a (Figure 5, A and B). In both cases, treatment with the *C3AR1* antagonist SB290157 significantly reduced the effect of C3a (Figure 5, A and B). Treatment with SB290157 similarly reduced the proliferation rate of HMC3 cells (Figure 5C). In line with these findings, primary murine macrophages also displayed increased expression of CD206 and CD163 upon treatment with C3a, while reducing the expression of the M1 marker CD86 (Figure 5, D–F). Effects of C3a on CD206 and CD86 were significantly reduced by treatment with SB290157 (Figure 5, D–F). Together, these data suggest that C3a promotes and M2-like phenotype of microglia and macrophages and that this effect at least partially is mediated through C3AR1. In murine GBM tissue, we found CD206^+^ cells exclusively in perivascular locations, as determined by costaining tumors for CD206 and the blood vessel marker CD31 (Figure 5G). Costaining of the panmacrophage marker F4/80 with C3 and the tumor cell marker Olig2 revealed that C3 expression itself was found in areas staining positive for both F4/80^+^ and Olig2^+^ cells (Figure 5H), indicating that C3 expression can conceivably come from both these cell types in murine GBM.

### C3aR is a potential therapeutic target in glioma.

**Figure 6:**
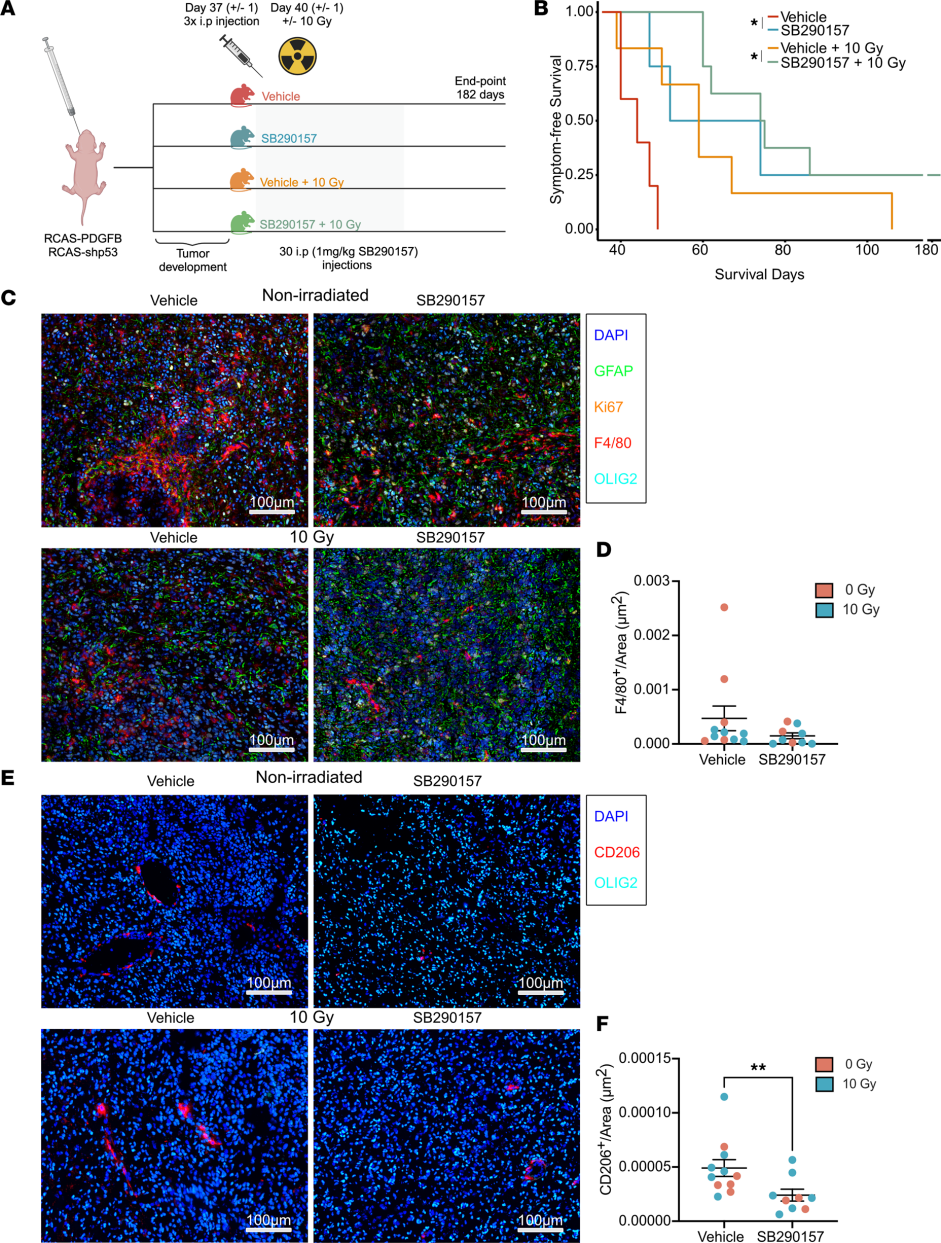
C3AR1 is a possible therapeutic target in GBM. (**A**) Illustration (created with BioRender.com) of study design for treatment with SB290157 (1 mg/kg) with or without combination with 10 Gy radiotherapy of murine gliomas induced through RCAS-PDGFB and RCAS-shp53 in Nestin/tv-a mice. (**B**) Kaplan-Meier curve showing the survival of mice treated with vehicle (red, *n* = 5), SB290157 (blue, *n* = 4), vehicle + 10 Gy (orange, *n* = 6), or SB290157 + 10 Gy (green, *n* = 8) as indicated. (**C**) Representative image of immunofluorescence staining of macrophages/microglia (F4/80), tumor cells (Olig2), and astrocytes (GFAP) in mice treated as indicated. (**D**) Quantitative analysis of F4/80^+^ cells/area (μm^2^) as indicated (*n* = 11 for untreated and *n* = 9 for SB290157 treated). (**E**) Representative images of immunofluorescence staining of tumor cells (Olig2) and CD206^+^ macrophages/microglia in mice treated as indicated. (**F**) Quantitative analysis of CD206^+^ cells/Area (μm^2^) as indicated (*n* = 11 for untreated and *n* = 9 for SB290157 treated). *P < 0.05, **P < 0.01, or ***P < 0.001. Statistical analysis was performed using the Mann-Whitney *U* test. Scale bars: 100 μm.

To investigate C3a/C3aR signaling as a possible therapeutic target in GBM, we generated murine tumors as previously described (Figure 1F). Daily i.p. injections (5 days on, 2 days off) of 1 mg/kg of the C3aR antagonist SB290157 were initiated at the time of emergence of brain tumor symptoms, with or without radiotherapy (10 Gy) (Figure 6A). We found that mice treated with the C3aR antagonist alone displayed increased survival (Figure 6B), similarly to what was observed with radiotherapy alone (Figure 6B). Furthermore, mice treated with a combination of the C3aR antagonist and radiotherapy showed further increased survival time as compared with radiotherapy alone (Figure 6B). Immunostaining (Figure 6C) of treated tumors showed no difference in the expression of F4/80 (Figure 6D), Olig2 (Supplemental Figure 5A), GFAP (Supplemental Figure 4B), or Ki67 (Supplemental Figure 5, C and D) in SB290157-treated tumors. However, in line with in vitro data supporting a role for the C3a/C3AR1 axis in macrophage polarization, tumors treated with SB290157 displayed fewer M2-polarized CD206^+^ macrophages (Figure 6, E and F), despite the lack of significant differences in the total macrophage/microglia population (Figure 6D), further supporting that C3a-C3aR1 signaling can modulate macrophage polarization. Most CD206^+^ cells were located in perivascular tumor areas or in association with blood vessel–like structures both within and outside the tumor area and both with and without treatment with SB290157 (Figure 6E and Supplemental Figure 6, A and B). CD206^+^ cells did not seem affected by SB290157 treatment in areas outside the tumor or in nontumor-bearing mice (Supplemental Figure 6, A and B). No difference in the distribution of C3 expression was apparent in tumors upon treatment with SB290157 (Supplemental Figure 6, C–F), and notably, radiation itself did not affect *C3* or *C3AR1* expression in tumors or most cultured GBM cells (Supplemental Figure 7, A–G). Tumors treated with a combination of radiotherapy and SB290157 were less vascular in general, as measured by the amount of CD34^+^ cells, but no such difference was apparent in nonirradiated tumors (Supplemental Figure 5, E and F). Together, these data suggest that C3aR is a potential therapeutic target in the GBM microenvironment, targeting of which might affect the cellular composition of tumors in general and macrophage polarization states specifically.

## Discussion

GBM recurrence is driven by treatment-resistant cells, which in turn are popularly believed to be associated with certain tumor niches, such as hypoxic or perivascular niches (29). Much effort has been directed toward characterizing the tumor cell response to hypoxia, but stromal cell responses to hypoxia and their potential contribution to tumor progression remain relatively unexplored. We have previously shown that astrocytes adopt a reactive phenotype with tumor-promoting properties in response to hypoxia and irradiation. In this study, we show that cells of the GBM microenvironment upregulate the central protein of the complement system, C3, under hypoxic conditions. Presence of C3 during cellular stress, such as hypoxia or irradiation, led to increased survival of some glioma cells, supporting previous findings that C3 can act as a prosurvival factor during cellular stress (28, 30, 31). However, these effects were limited to certain glioma cells and conditions, suggesting that the main effects on tumor growth in vivo are related to C3-mediated effects on stromal cells. Specifically, in vitro and in vivo evidence suggested a role for the C3/C3AR1 axis in promoting M2 polarization of macrophages.

We demonstrated that complement pathway gene expression is almost exclusive to tumor areas enriched for hypoxia-inducible gene expression in human GBM. While it is likely that this correlation is driven largely by the fact that the majority of complement gene expression in gliomas is derived from tumor-associated macrophages and microglia, cell types known to be enriched in hypoxic tumor areas (32), our experimental work established that at least *C3* and *C3AR1* could be directly induced in numerous cell types when cultured under hypoxic conditions. These findings add another level of complexity to previous reports linking hypoxia with the complement system. Olcina et al. demonstrated that tumors harboring mutations in the complement pathway displayed elevated hypoxia signaling (33). Furthermore, several genes associated with the complement system have been shown to be regulated by hypoxia in various cancer cell lines (34, 35).

Complement activation is a hallmark of several cancers. In particular, generation of C3a and C5a fragments has been associated with a tumor- and metastasis-supportive microenvironment. It is thought that malignant cells hijack complement regulatory pathways, resulting in chronic early complement activation, immunosuppression, angiogenesis, proliferation, EMT, and protumorigenic signaling (36, 37), while managing to escape complement-mediated lysis by upregulating late complement inhibitory proteins. Although each cancer can exhibit its unique cocktail of complement regulatory proteins, we show here that cells expressing C3 in the GBM microenvironment also upregulated several components of the classical pathway and downregulated early complement inhibitors. These data could suggest early complement activation in areas of hypoxia, where cells expressing C3 may still be protected from detrimental effects of complement activation through upregulation of the late complement inhibitor CD59.

Therapeutic targeting of the complement system has been shown to be somewhat challenging, due to the risks associated with tissue damage or infection when complement is dysregulated (38). C3a/C3aR signaling has been shown to be of importance in general brain physiology, including in neural development (39) and response to hypoxic-ischemic injuries (40), and it is considered an important regulator of BBB integrity and the brain vascular network (41, 42). Importantly, C3a/C3aR signaling has been associated with stemness and properties such as migration, proliferation, and angiogenesis in several cancers (43). Due to its high expression in gliomas, we hypothesized and demonstrated that the C3a/C3aR-signaling axis could be a suitable treatment target in GBM. Our findings that radiotherapy combined with C3aR inhibition could extend the lifespan of glioma-bearing mice are in line with recent work demonstrating efficacy of combining radiotherapy with C3a inhibition in preclinical models of pancreatic cancer (44).

Previous studies have shown that tumor associated macrophages (TAMs) are a major population of the TME in gliomas and are key contributors to several aspects of glioma progression. Attempts to deplete or reeducate TAMs through CSF-1/CSF-1R inhibition have shown promising results in preclinical studies and are currently in clinical development (45). Pyonteck et al. showed that inhibition of CSF-1R resulted in a blockage of glioma progression by reducing the number of M2-polarized macrophages, while preserving the total amount of TAMs (46). Recent work highlighted a subset of F4/80^hi^CD115^hi^C3aR^hi^CD88^hi^ TAMs that are recruited to melanoma tumors through the NF-κB1/CSF1R/C3aR axis (47). Anti-CSF1R therapy resulted in reduced tumor volume, F4/80 infiltration, and proliferation of macrophages as well as blood vessel formation (47). Similarly, blocking of a subset of TAMs positive for *C3AR1*, *CXCR4*, and *CSFR1*, using either anti-C3a therapy or SB290157 treatment of B16 melanoma mice, resulted in reduced F4/80 infiltration as well as slowed tumor growth (48). In our glioma model, SB290157-treated tumors had fewer M2-polarized macrophages as measured by CD206 expression, but no general reduction in macrophage/microglia content, as measured by F4/80. In vitro experiments supported a direct role of the C3a/C3AR1 axis in regulating macrophage polarization. CD206^+^ macrophages were associated with perivascular niches of the tumors both with and without irradiation. SB290157-treated tumors displayed a trend toward lower proliferation of F4/80^+^ cells, and tumors treated with SB290157 in combination with radiotherapy were less vascular as measured by CD34 immunostaining. These data are in line with previous reports suggesting that therapies targeting CSF-1/CSF1R and C3a/C3aR result in altered TAM polarization, recruitment, proliferation, and altered vascularity through regulation of VEGF. Previous reports established that C3aR-deficient or SB290157-treated mice display reduced vascular density (49, 50) and that C3aR^+^ TAMs located in perivascular niches display an M2-like character and are associated with regulation of angiogenesis and tumor aggressiveness (47, 48, 51–53).

Taken together, our findings suggest that C3a/C3aR could be a viable therapeutic target in GBM, targeting of which could affect the immunosuppressive TME of GBM by modulating macrophage polarization states.

## Methods

### Sex as a biological variable.

Animal, cell line, and clinical samples representing both sexes were used.

### Generation of murine gliomas.

Gliomas were induced in Ntv-a mice by intracranially injecting RCAS-PDGFB– and RCAS-shp53–transfected chicken fibroblast DF-1 cells (ATCC CRL-12203, ATCC) in the neonatal brain, as previously described (54). Mice were monitored daily and sacrificed upon development of glioma symptoms. For C3aR-antagonist studies, mice were randomized into groups and treated daily (5 days on, 2 days off) with 1 mg/kg SB290157 trifluoroacetate (Tocris, 6860/5) i.p., starting at the occurrence of glioma symptoms (day 37 ± 1) for up to a total of 30 injections. Nontumor-bearing mice were injected with 1 mg/kg SB290157 or vehicle i.p. starting at day 37 ± 1 during 1 week on the same treatment schedule as tumor-bearing mice. Radiotherapy was performed after 3 days of injections of SB290157 at day 40 ± 1. Mice were sedated using isofluorane, and cranial radiotherapy was administered with a 10 mm field in 1 single fraction of 10 Gy on a 220 kV preclinical research platform (XenX, XStrahl Inc.).

### Cell culture and treatments.

Primary human astrocytes (3H Biomedical) were cultured in astrocyte medium (3H Biomedical) supplemented with 2% FBS and 1% PenStrep solution. U3082MG, U3065MG, U3020MG, and U3084MG glioma cells were obtained from HGCC (hgcc.se) and were cultured in HGC medium on laminin-coated plates as described previously (55). Cells were passaged using Accutase (Thermo Fisher Scientific). Glioma cell line U251MG (MilliporeSigma) and HMC3 (ATCC) microglia were cultured in DMEM (Corning) supplemented with 10% FBS (Biological Industries) and 1% PenStrep (Corning). Cells were cultured at 37°C in a humidified incubator containing 21% O_2_ and 5% CO_2_, unless otherwise stated. Hypoxia was generated in a Whitley H35 Hypoxystation (Don Whitley Scientific) or an InvivO_2_ 400 Hypoxia Workstation (Baker Ruskinn).

### Proliferation assay.

Cells were seeded at a density of 0.5 × 10^3^ to 1 × 10^3^ cells per well in a 96-well plate, incubated with 180 ng/mL serum-purified C3. C3 was purified from human EDTA plasma using 2 consecutive anion exchange columns Q-Sepharose and DE52 cellulose. C3 was 95% pure as judged by gel electrophoresis. Plates were incubated either at 21% or 0.1% O_2_ as previously described. Cell viability/cell growth was measured at 24, 72, and 120 hours by addition of WST-1 (Abcam, ab155902) to the medium. Plates were read at 450 nm in a Syngery 2 Plate reader (BioTek).

### Colony formation assay.

GBM cells were plated at 150–300 cells/well in a 6-well plate. At day 2, cells were irradiated with 3–4 Gy using a CellRad x-ray irradiator (Faxitron) or a Cix1 x-ray irradiator (Xstrahl), after 1 hour with or without addition of 180 ng/mL human serum–purified C3. Cells were then allowed to incubate until visible colonies were formed or up to 14 days. Colonies were fixed in 4% PFA, followed by Crystal Violet 0.01% staining for 1 hour, and imaged in LAS-3000 system. Colonies were counted using Image J/Fiji (version 2.1.0).

### Immunofluorescence.

Human glioma cells were seeded on cover slip glass coated with poly-orthinine and laminin and allowed to attach. Cells were rinsed 3 times with phosphate-buffered saline (PBS) followed by addition of fresh medium with or without 180 ng/mL human serum–purified C3 and were placed at 0.1% oxygen for 72 hours. Cells were fixed in 4% paraformaldehyde (PFA) for 20 minutes followed by washing and permeabilization using 0.3% Triton X-100 (Sigma-Aldrich) in PBS. Cells were blocked using 2.5% fish gelatin in a solution of 0.05% Tween20 in PBS followed by incubation with primary anti-Ki67 (Thermo Fisher Scientific, rat, 14-5698-82), incubated overnight at 4°C. Cells were incubated with the appropriate secondary antibodies in the presence of Hoechst 33342 (Sigma-Aldrich) for 1 hour. Olympus BX63 microscope with DP80 camera and cellSens Dimension v 1.12 software (Olympus Corporation) were used to capture images. Ki67^+^ cells were quantified for each cell using QuPath.

### Multiplexed immunofluorescence.

Animals were euthanized upon glioma symptoms, and whole brains were embedded in Optimal cutting temperature (OCT) compound (Thermo Fisher Scientific) and frozen in ice-cold isopentane. Sections were air dried for 30 minutes followed by fixation in ice-cold acetone. Permeabilization was performed in 0.3% Triton X-100 in PBS. Sections were blocked in 1% BSA followed by primary antibody incubation.

To examine the C3 expression, we designed a custom multiplexed immunofluorescence detection panel using Alexa Fluor and antibodies targeting C3 (Hycult, HM1065, 1:150 dilution), Olig2 (R&D Systems, AF2418, 1:200 dilution) as a tumor marker, GFAP for detection of astrocytes (Abcam, ab4674, 1:400 dilution), and Nestin (Abcam, ab7659, 1:100 dilution) or CD31 (ProteinTech, 28083-1-A, 1.250) for detection of perivascular niches and the microglia/macrophage marker F4/80 (Abcam, ab111101, 1:100). After PBS wash, the following species-specific, fluorophore-conjugated secondary antibodies were used to reveal antibody staining (FITC [Abcam, ab63507], Alexa Fluor 594 [Abcam, ab150064], Alexa Fluor 647 [Invitrogen, A21247], and Alexa Fluor 555 [Abcam, ab150134]; 1:500 dilutions). Cell nuclei were labeled with DAPI (Sigma-Aldrich). To control for nonspecific signals, tissue sections were incubated with secondary antibodies alone. All antibodies were optimized for concentrations using monostainings imaged on an Olympus BX63 microscope. Scanning of the full antibody panel was performed on PhenoImager (Akoya Biosciences). A spectral fluorophore for each primary antibody and autofluorescence library was made to enable optimal multispectral unmixing. Spectral unmixing of images was performed using the inform software package (Akoya Biosciences).

Staining of brains from SB290157-treated mice was performed as described above, with Olig2, GFAP, Ki67 (Thermo Fisher Scientific, RM9106-S), and the microglia/macrophage marker F4/80 (Bio-Rad, MCA497) combined with the following secondary antibodies (FITC, Alexa Fluor 555, Alexa Fluor 594, and Alexa Fluor 647; 1:500). For vessel detection, staining was performed using CD31 (ProteinTech, 28083-1-A, 1.250) and CD34 (Thermo Fisher Scientific, 14-0341-81). M2-macrophages were labeled using CD206 (Invitrogen, MA5-16868).

### Quantitative PCR (qPCR).

RNA was isolated using the RNeasy Mini Kit together with the Qiashredder Kit (QIAGEN) according to the manufacturer’s protocol. cDNA was synthesized using random primers and Multiverse transcriptase enzyme (Applied Biosystems). Amplifications were run using a QuantStudio 7 real-time PCR system (Applied Biosystems) with SYBR Green Master Mix (Applied Biosystems). Melting curves were run for each primer pair to ensure specificity of primers. Primer sequences are listed in Table 2. Relative gene expression was normalized to the expression of 3 housekeeping genes (*SDHA*, *UBC*, and *YWHAZ*) using the comparative Ct method (56). All detections were performed in triplicates.

### Isolation of macrophages from mouse.

BM cells were collected from the femurs of Ntv-a mice by rinsing the bone cavities with DMEM. RBCs were lysed using a lysis solution (Miltenyi Biotec, 130-094-183), and macrophages were differentiated for 6 days with 20 ng/mL of mouse recombinant M-CSF (Peprotech, 315-02).

### Flow cytometry.

Primary mouse macrophages and HMC3 cells, treated with a combination of 10 μM SB290157, an equivalent amount of DMSO, and 100 nM C3a, were suspended in a stain buffer (BD Biosciences, 554657). To increase the specificity of labeling, cells were incubated with FcR blocking reagent (Miltenyi Biotec, 130-092-575) according to the manufacturer’s instructions. The purity of isolated macrophages was assessed using an anti-CD11b antibody (Immunotools, 22159116). The polarization of HMC3 cells was investigated with anti-CD206 (BioLegend, 321109) and anti-CD163 (R&D Systems, FAB1607A) antibodies. Mouse cells were stained with anti-CD206 (BioLegend, 141719), anti-CD163 (Abcam, ab313666), and anti-CD86 (BD Biosciences, 561962). The signal was measured using a FACSMelody flow cytometer (BD Biosciences), and the results were analyzed using FlowJo software (Tree Star Inc.).

### Sphere-forming assays.

For primary sphere formation, U3082MG cells were dissociated with Accutase and were then plated at single-cell density of 150 cells/well in a 6-well plate in 2.5 mL of HGC media. The spheres were grown until visible spheres were formed (up to 14 days) with control or treatments with 50 and 250 nM C3aR antagonist (Santa Cruz Biotechnology Inc., SB290157). For the ELDA, primary U3082MG spheres were dissociated and were then replated at 150–900 cells/mL in 300 μL in 8 wells of a 48-well dish followed by serial 1:2 dilution across 8 columns in the presence or absence of treatment until visible spheres were formed. Any well containing a sphere was scored as positive. Data combined from 4 biological repeats were quantified using the ELDA analysis tool (57).

### Patient cohort analysis.

Data from the Allen Institute for Brain Science IVY-GAP (http://glioblastoma.alleninstitute.org) (58) and the TCGA (TCGA_GBM and TCGA_LGG; ref. 59) were analyzed using the Gliovis data portal (60). Pan-TCGA data were analyzed using the Xena platform (61). The results published in here are in whole or part based upon data generated by the TCGA Research Network: https://www.cancer.gov/tcga Molecular signatures for HALLMARK_HYPOXIA (M5891) (62) and HALLMARK_COMPLEMENT (M5921) (62) in TCGA data were analyzed using GEPIA 2 (http://gepia2.cancer-pku.cn/#dataset) (62).

### 10x Visium spatial transcriptomics analysis.

Previously published human GBM spatial transcriptomic data sets (26) (available at https://datadryad.org/stash/dataset/doi:10.5061/dryad.h70rxwdmj) were processed according to the standardized pipeline as provided by Seurat (63) (https://satijalab.org/seurat/articles/spatial_vignette.html) in R. In short, data were normalized using SCTransform, and principal component analysis (PCA) and Uniform Manifold Approximation and Projection (UMAP) dimensionality reduction were all done using default parameters. We used the R package msigdbr (64) (version 7.5.1) to obtain the hypoxia and complement Hallmark gene sets and calculated average *Z* scores of the genes in each specific Hallmark signature in the spots of the GBM spatial transcriptomic Visium data. The Pearson correlation values (*P* values were corrected for multiple hypothesis testing using the Holm method) between the hypoxia and complement gene set expression scores in each spot were calculated using the R package correlation (65) (version 0.8.4). The hypoxia and complement *Z* scores were visualized onto the Visium tissue sections using Seurat’s SpatialFeaturePlot function, and the results from correlation analyses were visualized using the R package ggpubr (66) (version 0.6.0)

### Single-cell RNA-Seq analysis.

The processed single-cell RNA-Seq data set comprising 110 primary human GBMs (25) was downloaded from cellxgene (https://cellxgene.cziscience.com/collections/999f2a15-3d7e-440b-96ae-2c806799c08c). Dimensionality reduction coordinates and cell type annotations were as provided by the authors. All data were imported and analyzed using Seurat. Two-dimensional UMAPs of clusters, gene expression levels, and hypoxia and complement AddModuleScores (a function in Seurat to calculate gene set module scores) were visualized using the scCustomize package (67) (version 0.7.0) and the SCpubr package (68) (version 2.0.2). Seurat’s FindMarkers function (logfc.threshold = -Inf, min.pct = -Inf, min.diff.pct = -Inf) was used to obtain differentially expressed genes between the positive (expression > 0.0) and negative (expression ≤ 0.0) cell populations. For the GSEA, all genes were ranked by their estimated log_2_ fold changes. The analysis, as implemented in the fgsea package (69) (version 1.29.1), was performed using the Hallmark gene sets with 5% FDR and 1,000 permutations and visualized using ggplot2 (70) (version 3.4.4).

### Statistics.

Unless otherwise stated, all values represent mean ± SEM for at least 3 independent experiments. All statistical analyses were performed in GraphPad Prism version 9.3.1 or R-Studio version 2023.09.0+463. Statistical tests and number of replicates are indicated in Figure legends. Student’s *t* test were performed 2-tailed, and multiple comparisons were performed with 1-way ANOVA. Three levels of significance were used. A *P* value of less than 0.05 were considered significant.

### Study approval.

All animal procedures were conducted in accordance with the European Union directive about animal rights, and procedures were approved by Lund ethical committee (M-16123/19).

### Data availability.

The data that support the findings of this study are available in the Supporting Data Values file. Publicly available data sets can be accessed as described above. Materials can be obtained upon reasonable request by contacting the corresponding author.

## Author contributions

RR, KIS, JS, AMB, and AP designed the study. RR, KIS, EJ, CB, JW, VP, and CC conducted experiments. RR and JS acquired data. RR, KIS, JS, EJ, CB, JW, AMB, and AP analyzed data. CC, KP, AMB, and AP supervised the work. RR and AP drafted the manuscript. All authors reviewed, edited, and approved the final version of the manuscript.

## Supplementary Material

Supplemental data

Supporting data values

## Figures and Tables

**Table 2:**
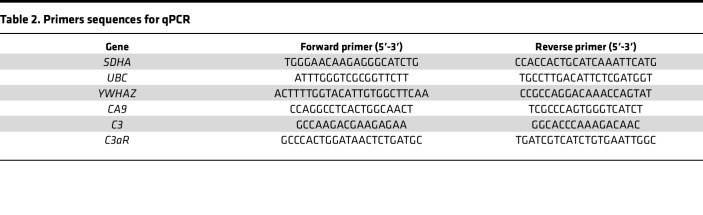
Primers sequences for qPCR

**Table 1:**
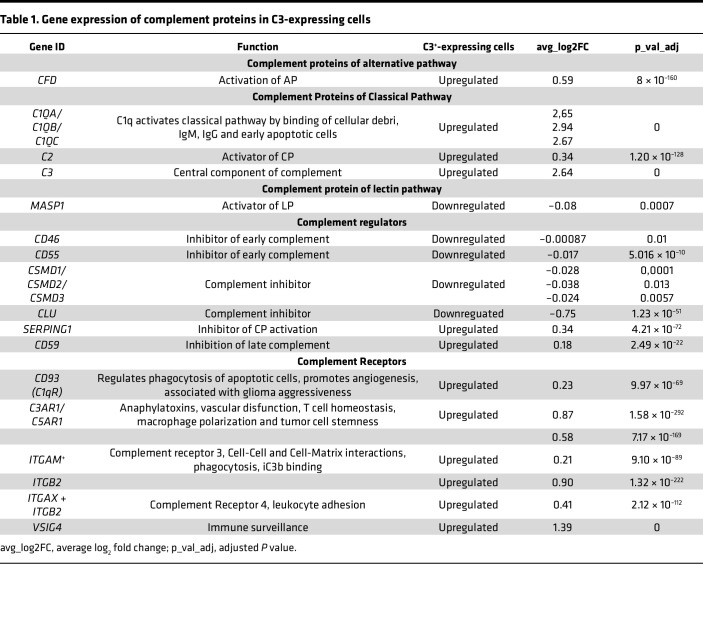
Gene expression of complement proteins in C3-expressing cells

## Version 1 08/22/2024

In-Press Preview

## Version 2 10/08/2024

Electronic publication
