# Supplementary material for: Hypoxia-induced complement component 3 promotes aggressive tumor growth in the glioblastoma microenvironment
Source: JCI Insight. 2024 Aug 22;9(19):e179854. doi: 10.1172/jci.insight.179854 (PMC11466187; doi:10.1172/jci.insight.179854)
Supplement: Supplemental data [file jciinsight-9-179854-s033.pdf]

Supplemental Data.

Rosberg et al., Hypoxia-induced Complement Component 3 Promotes Aggressive Tumor Growth in the Glioblastoma Microenvironment

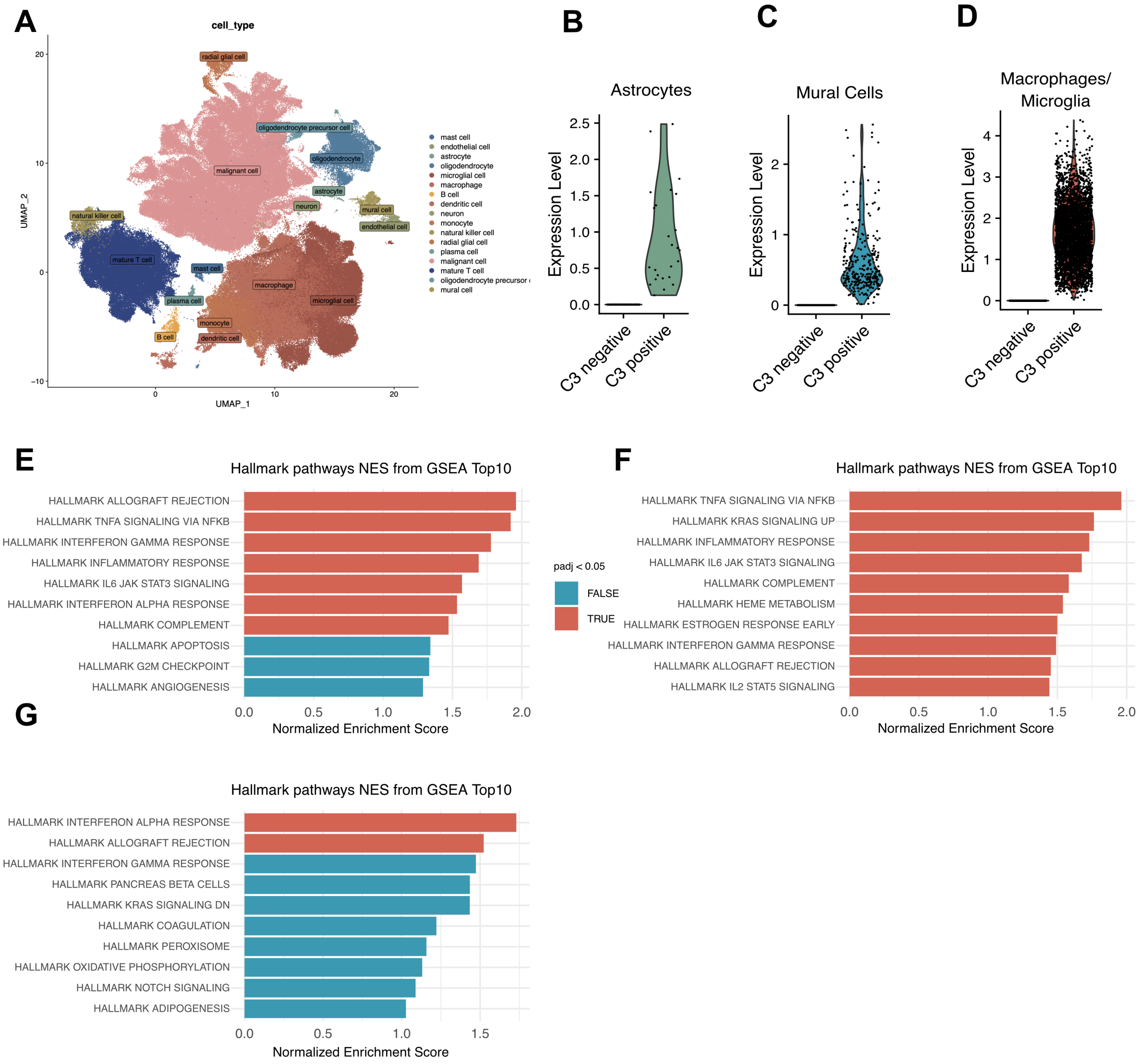

Supplemental Figure 1.

***A,** GBmap from Ruiz-Moreno, et al. 2022 (BioRxiv) comprising 16 data sets from 110 patients. **B-D**, C3 transcript level subdivided into C3-expressing or non-expressing cells from astrocytes, mural cells and macrophages/microglia. **E-G**, Hallmark enriched gene signatures in C3+ cells from astrocytes, mural cells and macrophages/microglia origin. Red- and blue-colored bars indicate significant and non-significant Benjamini-Hochberg adjusted P values (padj < 0.05), respectively.*

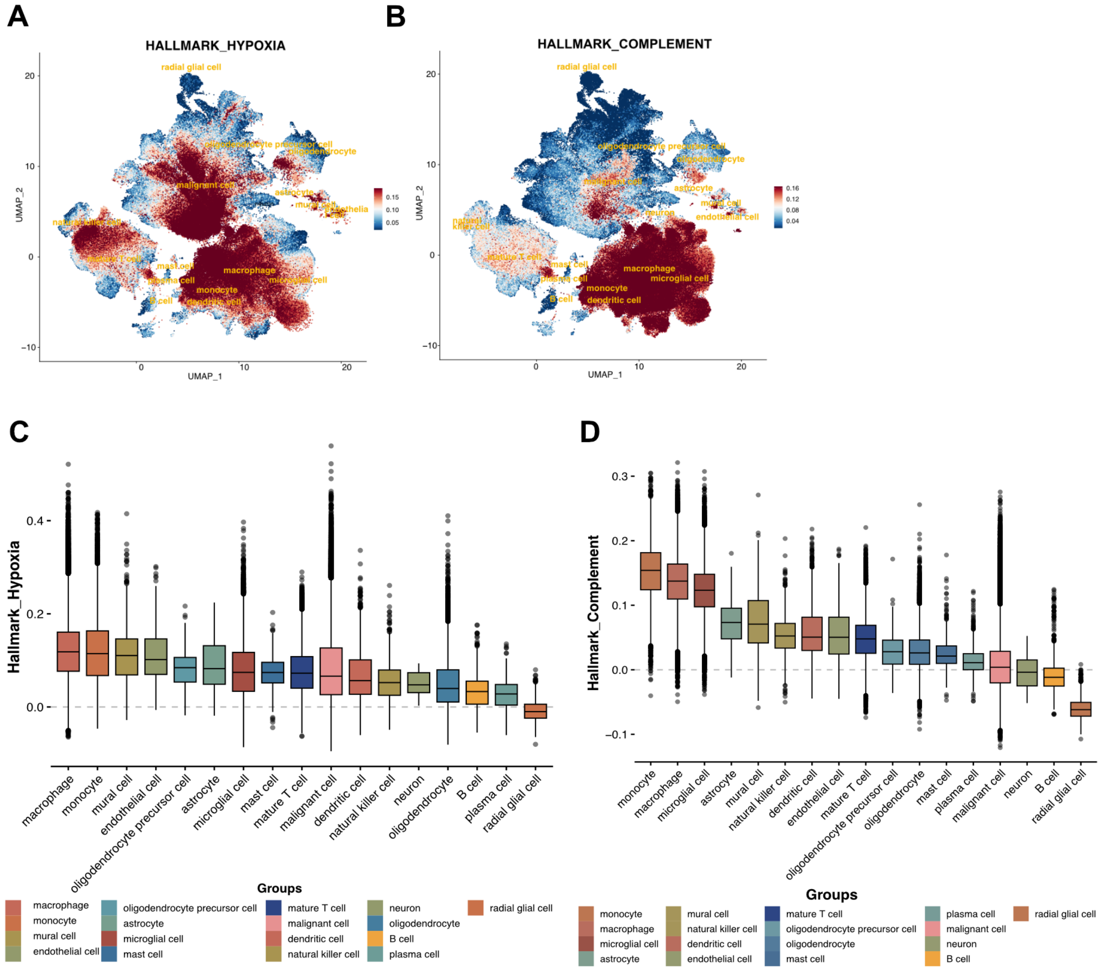

Supplemental Figure 2.
A, *UMAP displaying Hallmark hypoxia gene signature mapped onto GBmap.* ***B,*** *UMAP displaying Hallmark complement gene signature mapped onto GBmap.* ***C-D,*** *Cell types expressing hypoxic and complement gene signatures in GBM. Color scale in A-B and values in C-D indicate hypoxia and complement module scores.*

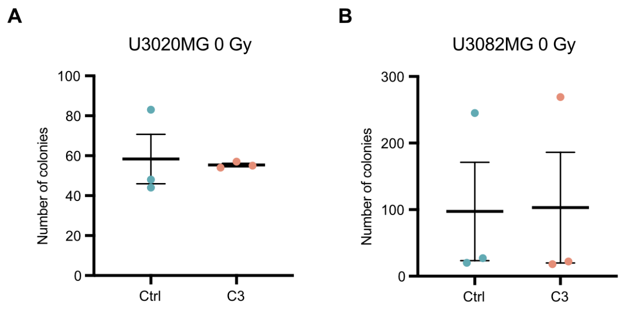

Supplemental Figure 3.
A-B. Quantification of number of colonies in U3020MG and U3082MG with or without presence of human C3 (180 ng/ml) in absence of irradiation.

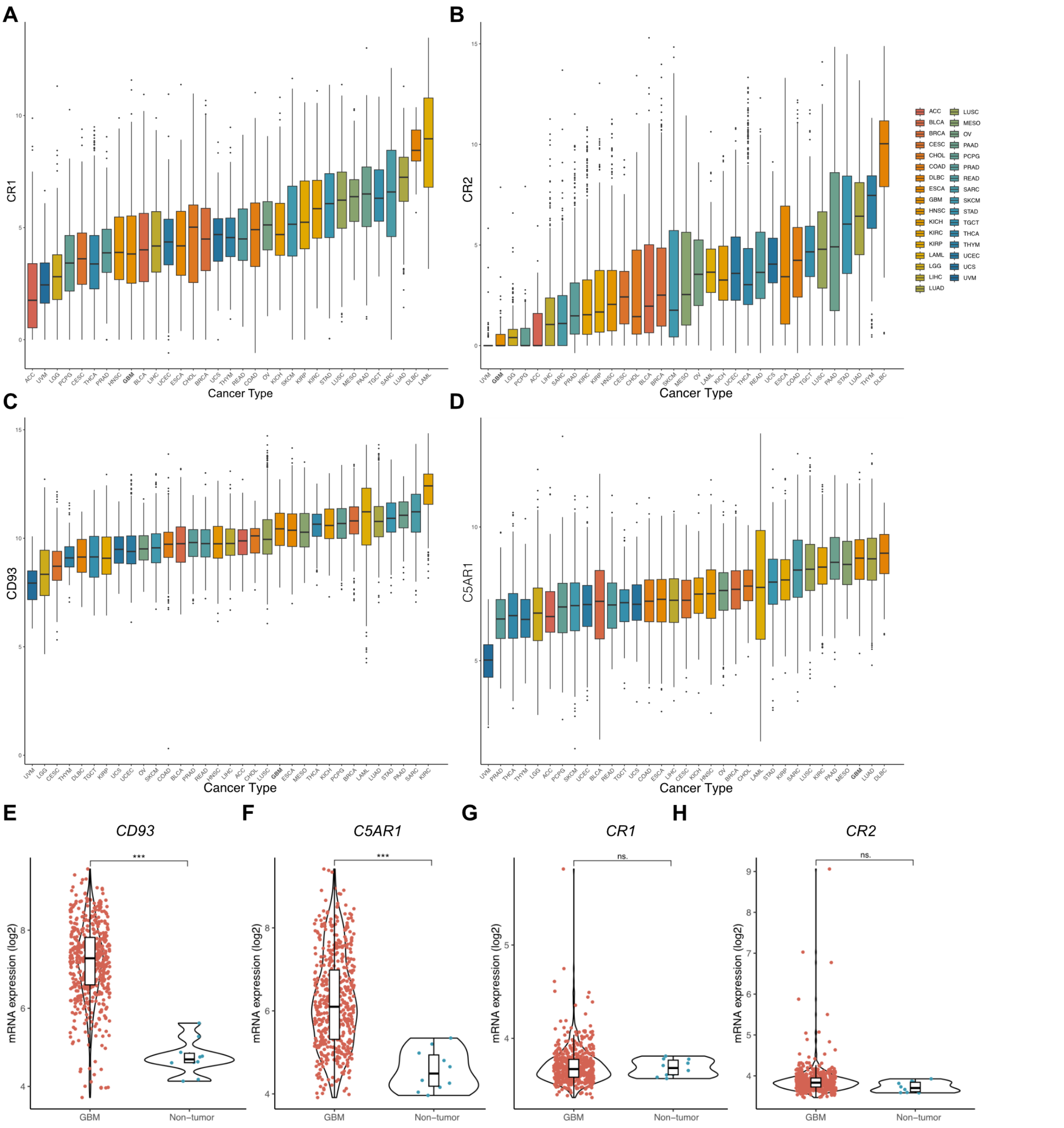

Supplemental Figure 4.

***A-D,** CR1, CR2, CD93 and C5AR1 transcript expression of Pan-Cancer TCGA data of common cancer types (n=33). **E-H,** CR1, CR2, CD93 and C5AR1 transcript expression in GBM compared to normal tissue. Statistical analysis were performed with Tukey's Honest Significant Difference (HSD).*

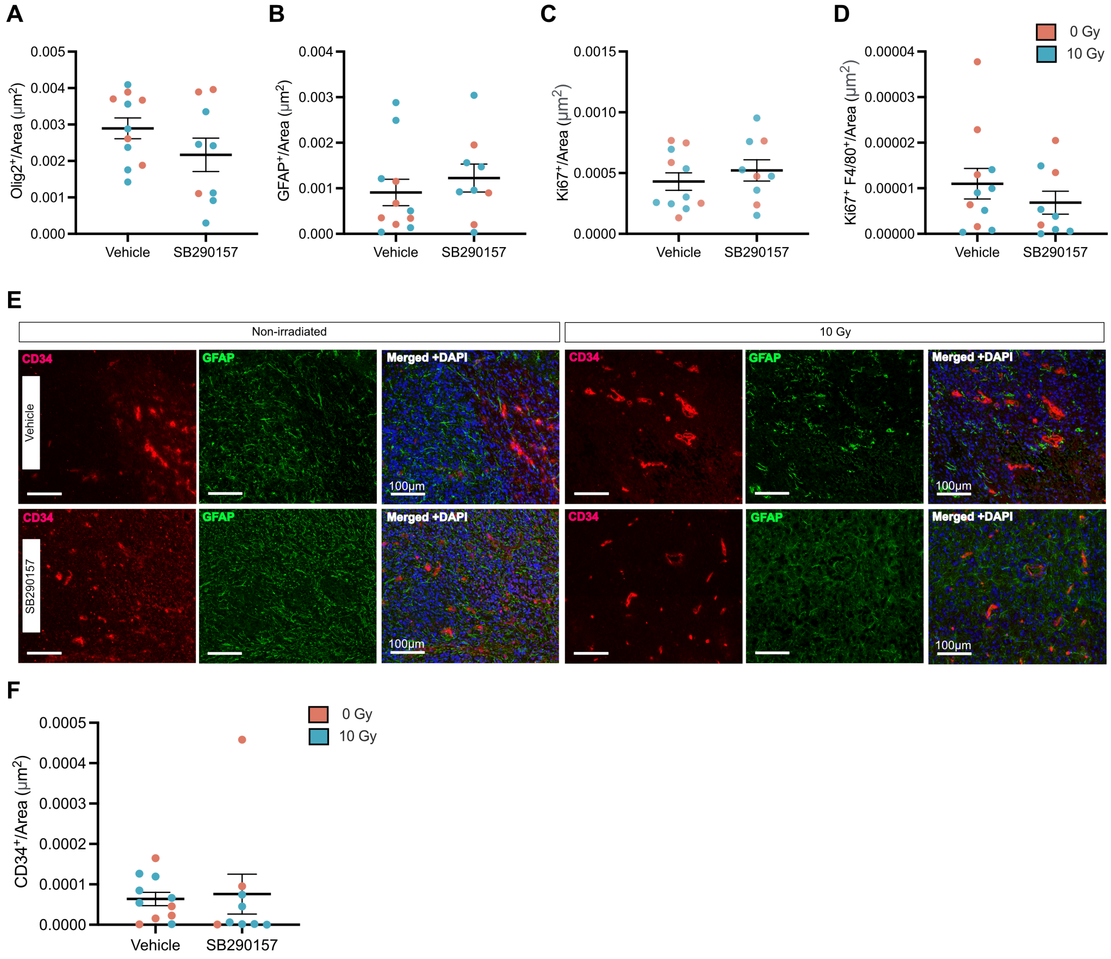

Supplemental Figure 5.
A-B, *Quantitative analysis of Olig2+ or GFAP+ cells/Area (μm^2) from Vehicle or SB290157 from immunofluorescent staining in figure 5C.* ***C,*** *Representative immunofluorescent staining of vessels (CD34) and astrocytes (GFAP) in SB290157 treated mice.* ***H,*** *Quantitative analysis of CD34+ cells/Area (μm^2) from Vehicle or SB290157. Statistical analysis were performed with Mann-Whitney U test for treatments. *, $P < 0.05$, **, $P < 0.01$, or ***, $P < 0,001$.*

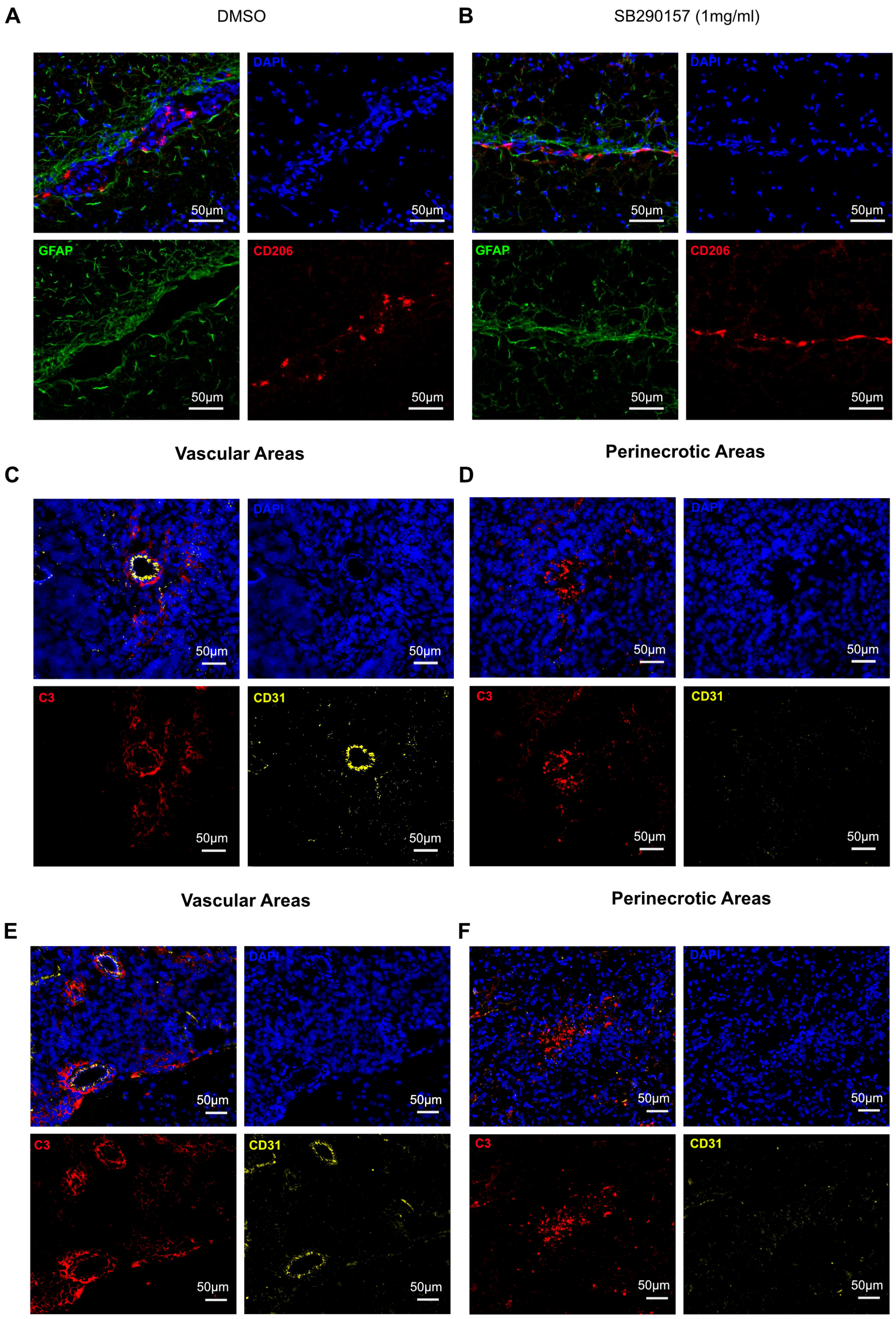

Supplemental Figure 6.
A-B. *Representative images of immunofluorescent staining of CD206+-cells in perivascular areas (CD31+) of non-tumor bearing mice injected with 1mg/ml SB290157 (n=4) or vehicle (n=4).* ***C-D.*** *Representative images of immunofluorescent staining of C3 in vascular and perinecrotic areas of SB290157 treated mice.* ***E-F.*** *Representative images of immunofluorescent staining of C3 in vascular and perinecrotic areas of 10 Gy + SB290157 treated mice.*

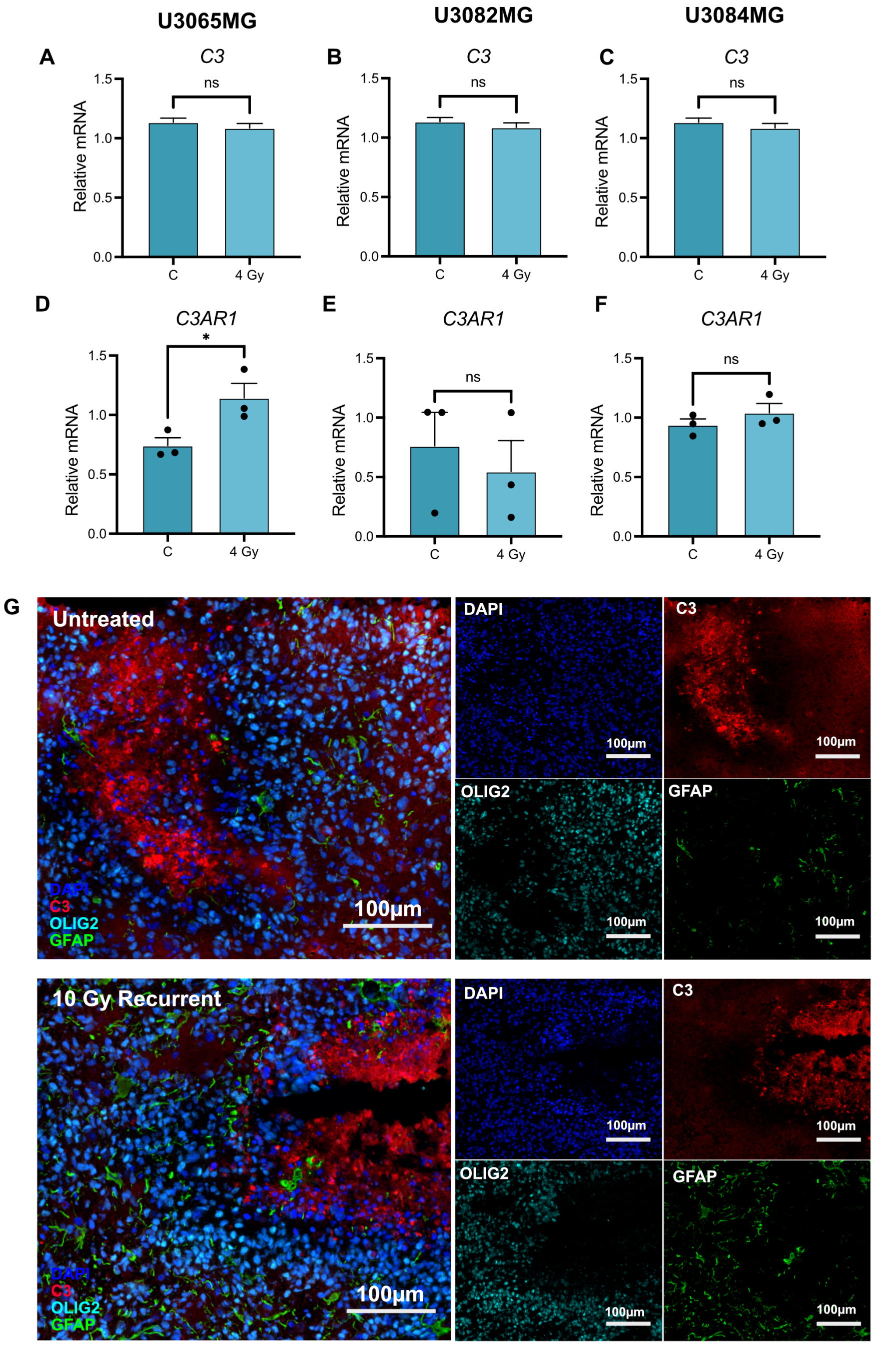

Supplemental Figure 7.
A-F. *Expression of C3 and C3AR mRNA in human primary glioma cells U3020 and U3082 in response to 3 Gy irradiation. Error bars represent SEM. *, P < 0.05, **, P < 0.01, or ***, P< 0,001. Statistical tests were unpaired t-test.* ***G.*** *Representative images of immunofluorescent staining of C3 from untreated or 10 Gy treated glioma bearing mice (n=5).*
